# PATIENTS WITH SOFT TISSUE SARCOMA AFTER TREATMENT BY NON ORTHOPEDIC ONCOLOGIC SURGEONS: EPIDEMIOLOGICAL PROFILE, STAGING, AND THERAPEUTIC CHALLENGES

**DOI:** 10.1590/1413-785220263401e293900

**Published:** 2026-02-13

**Authors:** Fernando Brasil do Couto, Eduardo Sadao Yonamine, Felipe Guimarães Magno, Luciano Elias Barboza, Vicente Magalhães de Araujo, Ana Beatriz Favacho Silva

**Affiliations:** 1 Hospital Ophir Loyola, Departamento de Oncologia Ortopedica, Belem, PA, Brazil.; 2 Santa Casa de Misericordia de Sao Paulo, Departamento de Oncologia Ortopedica, Sao Paulo, SP, Brazil.; 3 Hospital Metropolitano de Urgencia e Emergencia, Departamento de Ortopedia, Belem, PA, Brazil.

**Keywords:** Epidemiology, Neoplasm Staging, Bone Neoplasms, Sarcoma, Epidemiologia, Estadiamento de Neoplasias, Neoplasias Ósseas, Sarcoma

## Abstract

**Objective:**

this study analyzed patients with soft tissue sarcoma treated by non-orthopedic oncologists, evaluating their epidemiological profile, staging, and therapeutic challenges.

**Method:**

an analytical study in the form of a retrospective cohort, conducted through a review of medical records of patients treated at the hospital from January 1, 2011, to December 31, 2021.

**Results:**

a total of 61 patients were included, mostly male (55.7%), with a mean age of 42.8 years. The most frequent histological subtypes were synovial sarcoma (29.5%) and undifferentiated pleomorphic sarcoma (21.3%), with a predominance of high-grade tumors (75.4%). The majority of cases (77%) underwent resection, but without proper planning, leading to high recurrence rates (77%) and metastases (49.2%), with the lungs being the primary metastatic site. The mortality rate was 47.5%, with an average time to death of 3.1 years.

**Conclusion:**

the findings highlight the need for early diagnosis, specialized treatment, and multidisciplinary management to optimize clinical outcomes for patients. The adoption of standardized protocols in referral centers may reduce inadequate interventions and improve survival and quality of life for patients with soft tissue sarcoma. ***Level of Evidence III; Retrospective^f^ comparative study^e^***.

## RESUMO

**Objetivo:**

este estudo analisou pacientes com sarcoma de partes moles tratados por médicos não ortopedistas oncologistas, avaliando perfil epidemiológico, estadiamento e desafios terapêuticos.

**Método:**

estudo analítico em forma de coorte retrospectiva, realizado através de revisão de prontuários dos pacientes atendidos no hospital no período de 1 de janeiro de 2011 até 31 de dezembro de 2021.

**Resultado:**

foram incluídos 61 pacientes, majoritariamente homens (55,7%), com média de idade de 42,8 anos. Os subtipos histológicos mais frequentes foram sarcoma sinovial (29,5%) e sarcoma pleomórfico indiferenciado (21,3%), com predomínio de tumores de alto grau (75,4%). A maioria dos casos (77%) foi submetida à ressecção, mas sem planejamento adequado, resultando em elevadas taxas de recorrência (77%) e metástases (49,2%), sendo o pulmão o principal sítio metastático. O índice de mortalidade foi de 47,5%, com média de 3,1 anos para o óbito.

**Conclusão:**

os achados destacam a necessidade de diagnóstico precoce, tratamento especializado e manejo multidisciplinar para otimizar os desfechos clínicos dos pacientes. A adoção de protocolos padronizados em centros de referência pode minimizar intervenções inadequadas e melhorar a sobrevida e qualidade de vida dos pacientes com sarcoma de partes moles. ***Nível de Evidência III; Estudo retrospectivo^f^ comparativo^e^***.

## INTRODUCTION

Sarcomas are defined as a heterogeneous group of malignancies of mesenchymal origin, which can be divided into osteosarcomas, originating from bone tissue, and soft parts sarcoma (SPS), which arise from connective tissues. SPSs present more than 80 histological subtypes, with varying clinical presentations, leading to a difficult diagnosis.^1^

From an epidemiological point of view, SPSs have an incidence considered rare, representing about 1% of cancers in adult patients and 15% of pediatric cancers. However, it is believed that these data are underestimated, due to the pathogenesis not yet fully clarified and its challenging anatomopathological findings.^2^

Although there is no clearly defined pathogenesis, some risk factors have already been identified, such as genetic predisposition, immunodeficiency, lymphedema, and history of exposure to oncogenic viruses, radiation, chemotherapy and/or chemical carcinogens. In addition, certain genetic syndromes have a well-characterized association with SPS, such as Li-Fraumeni syndrome, Bloom syndrome, Beckwith-Wiedemann syndrome, hereditary retinoblastoma and neurofibromatosis type 1.^3^

The anatomical location of the disease is a significant variable, with great influence on the patient's treatment and prognosis. The upper and lower extremities account for 43% of cases, corresponding to the most common primary site. However, tumors can develop anywhere in the body, such as the viscera (19%), retroperitoneum (15%), trunk (10%), and head and neck (9%).^4^

SPSs can occur at any time in the patient's life, with histological variations according to the age group in which their evolution occurs. When all ages are considered, the most common forms include: liposarcoma; indifferent pleomorphic sarcoma, sinovial sarcoma and leiomiosarcoma. In the elderly, mixfibrosarcoma also gains importance. In childhood, rabdomiosarcoma is the main representative, causing 50% of cases.^5^

Despite the presence of small variations according to each histological subtype, the main clinical form associated with SPS is the emergence of a painless nodular lesion, which can last for weeks or months. Less commonly, they present pain, pathological fractures, or are discovered incidentally. This non-specific picture contributes to the difficulty of making the proper diagnosis.^6^

Some characteristics associated with the tumor may indicate to the responsible doctor the need for a more urgent investigation. The existence of a painless mass with progressive increase in size is the main alarm sign for an increased risk of malignancy. In addition, lesions with a length greater than 5 centimeters, or of more hardened consistency than the surrounding tissue, or of deep location relative to the fascia, require more cautious investigation.^7^ Histopathological diagnosis remains the gold standard for identification of the subtype and proper conduct of the case. However, it is worth noting that differentiating between malignant and benign conditions can be extremely complicated, requiring experienced pathologists for a reliable judgment.^8^

The complete staging includes imaging of the primary tumor and verification of the presence of metastatic disease. Magnetic resonance imaging is the modality of choice to define the size of the tumor, as well as its muscular involvement and the relationship with neurovascular structures. Considering that the most common places of metastasis appearance are the lungs, thoracic CT gains importance for confirmation or discard.^9^

The main method of treatment for SPS is surgical, with extensive resection, removing enough tissue to reach macroscopically negative margins, with the objectives of preventing local recurrence and preserving limb function. In the case of highly infiltrative tumors, radiation therapy, neoadjuvant or adjuvant, may be necessary for better control of the margins. Adjuvant chemotherapy gains importance for therapeutic aid in metastatic diseases.^10^

The main risk of death related to SPSs of extremities is the occurrence of metastases. The most relevant prognostic factors for overall survival are: age of the patient; tumor dimensions; degree of malignancy; histological subtype; and depth. In addition, local recurrence is a constant danger associated with SPS, contributing to a worse patient outcome.^11^

Therefore, considering the complexity involving the different pathological forms of SPS, it is crucial to recognize the importance of managing such cases in specialized units. Driving by experienced teams with the help of appropriate technologies is linked to greater overall survival of patients, as well as higher rates of surgeries with negative margins and ease of receiving adjuvant therapy.^12^

## METHODOLOGY

This study is descriptive, retrospective and observational. The research was conducted in the oncological orthopedic sector of Ophir Loyola Hospital, linked to the Government of the State of Pará, located in the Municipality of Belém, State of Pará.

The target population of this study consists of patients diagnosed with soft part sarcoma and treated by non-orthopedic oncologists, who are subsequently referred to Ophir Loyola Hospital for specialized treatment in the sector of orthopedic oncology.

The sample of this study was carefully selected to adequately represent the target population. Patients registered in the Ophir Loyola Hospital system were selected between 2011 and 2021. The sample size was determined considering the availability of eligible patients and the available resources for data collection and analysis, totalling 61 patients selected.

It was considered as inclusion criteria to have a confirmed histopathological diagnosis of soft-part sarcoma, with prior treatment by non-orthopedic oncologists, aged over 12 years and availability of medical records.

The exclusion criteria consisted of: patients who received treatment exclusively by oncological orthopedists, without a confirmed histopathological diagnosis of soft-part sarcoma, who were younger than 12 years of age and/or unavailable or insufficient medical records. The data collection for this study was conducted in a systematic and comprehensive manner, in accordance with ethical protocols and data privacy regulations, ensuring the confidentiality and anonymity of patients.

The data was obtained by reviewing the medical records of the patients included in the study to collect detailed information about demographic profile, medical history, imaging examinations, tumor characteristics, received treatments and clinical outcomes.

The data analysis for this study was conducted in a thorough and rigorous manner, using statistical and epidemiological methods suitable for each specific purpose of the research.

Initially, the evaluation of the studied population was carried out, with identification of the epidemiological profile, staging and corresponding therapeutic challenges. These data were tabulated in a spreadsheet elaborated in the Microsoft® Office Excel® 2016 software, going through descriptive statistical analysis of the sample characterization, with frequency, percentages, average, standard deviation, median, interquartile interval (p25%-p75%), exposed in tables and/or graphs. The continuous quantitative variables, such as age (years) and time (years), were first submitted to the Shapiro-Wilk test to analyse their normality distribution. All statistical analysis was performed in SPSS 20.0 software.

This research was approved by the Ethics Committee of the Institution, through the Brazil Platform, with identification CAEE 81512924.5.0000.5550 (amendment no. 7.059.611). The collection of records in a retrospective manner was preceded by a Data Usage Commitment Terms, to ensure the reliability of the collected information, and the Request for waiver of the Free and Informed Consent Terms.

## RESULTS

The study included 61 subjects, mostly male (55.7%), with an average age of 42.8 (±19.4) years, brown (62.3%), 34.2% with some comorbidity, among the most frequent hypertension (29.5%) and diabetes (23.0%), as can be seen in Table 1.

**Table 1:** Demographic and epidemiological profile of patients with soft-part sarcoma treated by non-orthopedic oncologists.

Variables	Frequency (n. 61)	Percentage (%)	CI95%
**Sex**			
Male	34	55.7	44.3 - 67.2
Female	27	44.3	32.8 - 55.7
**Age (years)**			
Average (±SD)	42.8 (±19.4)		38.1 - 48.0
Median (p25%-75%)	36.0 (28.0 - 60.0)		33.0 - 46.0
**Ethnicity**			
Black	15	24.6	13.1 - 36.0
Pardo	38	62.3	49.2 - 75.4
White	8	13.1	4.9 - 23.0
**Comorbidities**			
Hypertension	18	29.5	19.7 - 41.0
Diabetes	14	23.0	13.1 - 34.4
Colelithiasis	2	3.3	0.0 - 8.2
Benign prostatic hyperplasia	2	3.3	0.0 - 8.2
Encephalic Vascular Accident	1	1.6	0.0 - 4.9

CI. Confidence interval. SD. standard deviation. p. percentile.

Table 2 presents the clinical profile of 61 patients with soft-part sarcoma treated by non-orthopedic oncologists. In terms of anatomical location, the thigh was the most affected region, representing 52.5% of the cases (IC95%: 41.0 – 65.6). Other locations, such as leg (11.5%; IC95%): 4.9 - 19.7), hand and forearm (9.8% each; 95% CI: 3.3 – 18.0), arm (8.2%; IC95%: 1.6 - 14.8), foot (6.6%; 95% CI: 1.6 - 13.1) and handle (1.6%; IC95%: 0.0 – 4.9), were less common. The most common histological subtypes were sinovial sarcoma, with 29.5% of cases (95% CI: 18.0 – 42.6), and indifferent pleomorphic sarcoma, with 21.3% (CI95%: 11.5 – 31.1). Other subtypes, such as liposarcoma and epithelial sarcoma (9.8% each), rabdomiosarcoma and leiomiosarcoma (8.2% each), as well as less prevalent types, such as angiosarcoma, dermatofibrossarcoma protuberans, fibrosarcoma, mixfibrosarcoma, neurofibrosarcoma and alveolar sarcoma (1.6% to 3.3%), presented a lower incidence.

**Table 2:** Clinical profile of patients with soft part sarcoma treated by non-orthopedic oncologists.

Variables	Frequency (n. 61)	Percentage (%)	CI95%
**Anatomic Localization**			
Arm	5	8.2	1.6 - 14.8
Frontarm	6	9.8	3.3 - 18.0
Hand	1	1.6	0.0 - 4.9
Hand	6	9.8	3.3 - 18.0
Butt	32	52.5	41.0 - 65.6
Leg	7	11.5	4.9 - 19.7
Foot	4	6.6	1.6 - 13.1
**Histological subtype**			
Angiossarcoma	1	1.6	0.0 - 4.9
Dermatofibrosarcoma protuberans	2	3.3	0.0 - 8.2
Fibrossarcoma	2	3.3	0.0 - 8.2
Leiomiossarcoma	5	8.2	1.6 - 16.4
Liposarcoma	6	9.8	3.3 - 18.0
Mixfibrossarcoma	1	1.6	0.0 - 4.9
Neurofibrossarcoma	1	1.6	0.0 - 4.9
Rabdomiossarcoma	5	8.2	1.6 - 14.8
Sarcoma alveolar	1	1.6	0.0 - 4.9
Epithelial sarcoma	6	9.8	3.3 - 18.0
Undifferentiated pleomorphic sarcoma	13	21.3	11.5 - 31.1
Sinovial sarcoma	18	29.5	18.0 - 42.6
**Grade**			
Low	9	14.8	64.0 - 85.2
Moderate	6	9.8	3.3 - 18.0
High	46	75.4	64.0 - 85.2
Procedures			
Resection	47	77.0	65.6 - 86.9
Biopsia	14	23.0	13.1 - 34.4

CI. Confidence interval.

As for the degree of sarcoma, 75.4% of cases were of high degree (CI95%: 64.0 – 85.2), while low and moderate degrees were observed in 14.8% and 9.8% of patients, respectively. Regarding the procedures performed, the majority of patients (77%; 95% CI): 65.6 – 86.9) was subjected to resection, while biopsy was performed in 23% of cases (CI95%: 13.1 – 34.4).

When analysing the treatments performed by Ophir Loyola Hospital for patients including, it was observed that these patients began follow-up in the reference unit about 1.1 (±2.2) years after the diagnosis of the disease, and the vast majority underwent chemotherapy (77.0%) and radiotherapy (60.7%), as evidenced in Table 3. Table 4 presents a detailed analysis of recurrence, metastases and deaths among 61 patients with soft-part sarcoma previously treated by non-orthopedic oncologists. Regarding recurrence, 77% of patients had recurrence (CI95%: 67.2 - 86.9), with an average recurrence time of 1.7 years (±1.2; 95% CI: 1.4 – 2.0). Only 23% of patients do not had recurrence (CI95%: 13.1 – 32.8).

**Table 3:** Treatment carried out by Ophir Loyola Hospital of patients with soft-part sarcoma treated by previous non-orthopedic oncologists.

Variables	Frequency (n. 61)	Percentage (%)	CI95%
Time to start the treatment			
Average (±SD)	1.1 (±2.2)		0.5 - 1.6
**Treatment performed**			
Resection	29	47.5	34.4 - 59.0
Amputation	23	37.7	26.2 - 50.8
Chemotherapy	47	77.0	67.2 - 86.9
Radioterapia	37	60.7	49.2 - 72.1

CI. Confidence interval. dp. standard deviation.

**Table 4:** Analysis of recurrence, metastases and deaths of patients with soft part sarcoma treated by previously non-orthopedic oncologists.

Variables	Frequency (n. 61)	Percentage (%)	CI95%
**Recurrence**			
Yes	47	77.0	67.2 - 86.9
No.	14	23.0	13.1 - 32.8
Time for Recurrence (years)	1.7 (±1.2)		1.4 - 2.0
**Metastasis**			
Yes	30	49.2	36.1 - 62.3
No.	31	50.8	37.7 - 63.9
Time for Metastasis (years)	2.4 (±1.6)		1.9 - 3.0
**Sites of metastases**			
Lung	25	41.0	27.9 - 54.1
Vertebral Spine	8	13.1	4.9 - 22.9
Axilar lymph nodes	2	3.3	0.0 - 8.2
English lymph nodes	4	6.6	1.5 - 13.1
Cranium	5	8.2	1.6 - 16.4
Liver	4	6.6	1.5 - 13.1
Costa Arch	1	1.6	0.0 - 4.9
Adrenal	1	1.6	0.0 - 4.9
**Death**			
Yes	29	47.5	34.4 - 60.7
No.	32	52.5	39.3 - 65.6
Pre-Treatment Death	6	9.8	3.3 - 18.0
Time for Death (years)	3.1 (±1.8)		2.4 - 3.7

CI. Confidence interval. SD. standard deviation.

As for metastases, 49.2% of patients developed metastases (95% CI: 36.1 – 62.3), with an average time to appearance of 2.4 years (±1.6; 95% CI: 1.9 – 3.0). The lung was the most common site of metastases, affecting 41% of cases (IC95%: 27.9 – 54.1), followed by the spine (13.1%; 95% CI: 4.9 – 22.9), skull (8.2%; IC95%: 1.6 – 16.4), inguinal lymph nodes and liver (6.6% each; 95% CI: 1.5 – 13.1). Other localizations, such as axillary, coastal and adrenal lymph nodes, were less common, ranging from 1.6% to 3.3%.

On deaths, 47.5% of patients died (IC95%: 34.4 – 60.7), while 52.5% survived (IC95%: 39.3 – 65.6). Among deaths, 9.8% occurred prior to treatment (IC95%: 3.3 – 18.0). The mean time to death was 3.1 years (±1.8; 95% CI: 2.4 – 3.7).

In general, the results reveal that patients with soft-part sarcoma treated by non-orthopedic oncologists, the majority were male (55.7%), with an average age of 42.8 years, predominating brown (62.3%). The most common comorbidities were hypertension (29.5%) and diabetes (23.0%). The thigh was the most frequent anatomical location (52.5%), followed by leg (11.5%) and forearm/hand (9.8% each). The main histological subtypes were sinovial sarcoma (29.5%) and indifferent pleomorphic sarcoma (21.3%). Most tumors were of high degree (75.4%) and were treated with resection (77%). Metastases occurred in 49.2% of cases, with the lungs being the main site (41%). Recurrence was observed in 77%, with an average time span of 1.7 years. Deaths occurred in 47.5%, with an average of 3.1 years to the outcome.

## DISCUSSION

The epidemiological profile of soft-part sarcoma indicates that this is a rare condition, corresponding to approximately 1% of all cancers. The prevalence is slightly higher among men than women, according to the results found. Most cases are observed among the black population, while cases among whites are less frequent. The significant predominance of cases among pardos, identified in the research, can be attributed to the ethnic differences present in the Amazon region.^13^

Regarding the age group, the results found a comprehensive range, from 14 to 99 years, with the predominance of cases in older patients, in accordance with the standard epidemiological profile of soft-part sarcomas. However, there was a difference between the average age identified by the work, 42.8 years, and the main literature, 60 years. This finding may be a reflection of inadequate interventions during early stages of the disease, accelerating the development of lesions.^14^

Soft-part sarcomas can develop in various areas of the body, but there is a preference for specific locations. The extremities, especially the lower limbs, are the regions most frequently affected, which is in accordance with the results of the study, which showed a significant predominance of cases with the primary site in the thigh and leg.^15^ The large histological variety identified in this study is consistent with what is described in the literature, which has already documented more than 80 distinct subtypes of soft-part sarcomas. In addition, the most common histological types include liposarcoma, indifferent pleomorphic sarcoma, sinovial sarcoma and leiomiosarcoma, corroborating the observed results. However, the high frequency of high-grade subtypes may be the result of inadequate initial management.^5^

Regarding the initial treatment performed by non-specialists, the study revealed inadequate interventions in most cases, such as incomplete resections and the absence of adequate adjuvant therapies. Since it is a heterogeneous group of rare neoplasms, soft-part sarcomas should be accompanied by an experienced multidisciplinary team, ideally composed of a radiologist, pathologist, radiotherapist, oncologist and oncological orthopedist, in order to obtain the best standard of care.^16^

In addition, only 23% of patients treated by non-orthopedic oncologists underwent biopsy, which is fundamental not only for histopathological diagnosis, but also for the planning of appropriate therapy. The absence of this procedure increases the risk of lesions with positive margins and subtreatment, reinforcing the relevance of standardized diagnostic protocols in specialized centers.^4^

The main objectives in the treatment of soft-part sarcoma are to ensure patient survival, prevent recurrence, preserve function and reduce morbidity, with emphasis on surgical treatment as the main therapeutic approach. However, for the treatment to be effective, it is essential to perform a wide resection with free margins, which may not have been achieved in the initial treatment carried out by professionals who are not specialized in orthopedic oncology.^17^

In addition, 37% of cases have evolved to amputation, which is considered an extreme measure for patients with bulky tumors, involvement of the neurovascular beam and dysfunctional limbs. Although amputation should not be considered a therapeutic failure, it is worth considering the possibility of a positive outcome with the application of appropriate therapies.^3^

Recurrence of soft-part sarcoma is strongly associated with high histological degree, which corroborates the findings of this study. However, the recurrence rate of 77% over an average period of 1.7 years differs considerably from the average range of 17-26% after 5 years. This discrepancy may suggest the relevance of early diagnosis and proper treatment to reduce recurrence rates.^9^

During optimal treatment, up to 40% of patients with soft-part sarcoma develop metastatic disease. However, the study confirmed the occurrence of 49.2% of metastases in cases treated by non-orthopedic oncologists. This reinforces the importance of specialized follow-up for achieving the best possible outcome. Regarding the most common locations, the prevalence of lung and vertebral metastases is consistent with the epidemiology.^18^

The deaths observed among the analysed patients demonstrate the severity associated with the management of soft-part sarcomas. The death rate of 47.5%, with an average of 3.1 years to the outcome, represents an unfavorable finding compared to the global, of 35% in 5 years. The high prevalence of high-grade histological subtypes, the underuse of adjuvant therapies and the inappropriate conduct of non-specialists may have contributed to a worse result in this study.^19^ In view of the above, soft-part sarcomas represent a significant challenge in diagnosis and treatment due to their rarity, histological diversity and the complexity involved in their management. The findings of this study highlight the importance of early diagnosis, specialist treatment and multidisciplinary approach to improve outcomes. The high rate of mortality and recurrence observed, as well as the evolution to metastases in many cases, highlights the urgent need for more effective diagnostic protocols, as well as the implementation of appropriate therapies from the start of treatment.

## Data Availability

The data set for this article is available in the Figshare digital repository: https://doi.org/10.6084/m9.figshare.30698471

## References

[1] Hall F, Villalobos V, Wilky B (2019). Future directions in soft tissue sarcoma treatment. Curr Probl Cancer.

[2] Moreira SBR, Oliveira RFF, Baptista TS, Lima LC, Forte ECN (2022). Sarcoma - characteristics and results in a reference oncology center in southern Brazil. Brazilian Journal of Health Review.

[3] Brownstein JM, DeLaney TF (2020). Malignant Soft-Tissue Sarcomas. Hematol Oncol Clin North Am.

[4] von Mehren M, Kane JM, Agulnik M, Bui MM, Carr-Ascher J, Choy E (2022). Soft Tissue Sarcoma, Version 2.2022, NCCN Clinical Practice Guidelines in Oncology. J Natl Compr Canc Netw.

[5] Nakayama R, Mori T, Okita Y, Shiraishi Y, Endo M (2020). A multidisciplinary approach to soft-tissue sarcoma of the extremities. Expert Rev Anticancer Ther.

[6] Moten AS, Zhao H, Howell K, Nadler A, Reddy SS, von Mehren M (2019). Soft tissue sarcoma of the extremity: Characterizing symptom duration and outcomes. Surg Oncol.

[7] Ardakani AHG, Woollard A, Ware H, Gikas P (2022). Soft tissue sarcoma: Recognizing a rare disease. Cleve Clin J Med.

[8] Bourcier K, Le Cesne A, Tselikas L, Adam J, Mir O, Honore C (2019). Basic Knowledge in Soft Tissue Sarcoma. Cardiovasc Intervent Radiol.

[9] Roland CL (2020). Soft Tissue Tumors of the Extremity. Surg Clin North Am.

[10] Jakob J, Schmolders J (2019). Systematic planning of surgery for soft tissue sarcoma of the extremities. Chirurg.

[11] Spolverato G, Callegaro D, Gronchi A (2020). Defining Which Patients Are at High Risk for Recurrence of Soft Tissue Sarcoma. Curr Treat Options Oncol.

[12] Lazarides AL, Kerr DL, Nussbaum DP, Kreulen RT, Somarelli JA, Blazer DG (2019). Soft Tissue Sarcoma of the Extremities: What Is the Value of Treating at High-volume Centers?. Clin Orthop Relat Res.

[13] Cosci I, Del Fiore P, Mocellin S, Ferlin A (2023). Gender Differences in Soft Tissue and Bone Sarcoma: A Narrative Review. Cancers (Basel).

[14] Crombé A, Kind M, Fadli D, Miceli M, Linck PA, Bianchi G (2023). Soft-tissue sarcoma in adults: Imaging appearances, pitfalls and diagnostic algorithms. Diagn Interv Imaging.

[15] Kask G, Repo JP, Tukiainen EJ, Blomqvist C, Barner-Rasmussen I (2021). Soft Tissue Sarcoma of Lower Extremity: Functional Outcome and Quality of Life. Ann Surg Oncol.

[16] Gamboa AC, Gronchi A, Cardona K (2020). Soft-tissue sarcoma in adults: An update on the current state of histiotype-specific management in an era of personalized medicine. CA Cancer J Clin.

[17] Rothermundt C, Andreou D, Blay JY, Brodowicz T, Desar IME, Dileo P (2023). Controversies in the management of patients with soft tissue sarcoma: Recommendations of the Conference on State of Science in Sarcoma 2022. Eur J Cancer.

[18] Spalato-Ceruso M, Ghazzi NE, Italiano A (2024). New strategies in soft tissue sarcoma treatment. J Hematol Oncol.

[19] Anderson AB, Park AB, Zhu K, Lin J, Shriver CD, Potter BK (2022). Soft-tissue Sarcoma Survival in the US Military Health System: Comparison With the SEER Program. J Am Acad Orthop Surg Glob Res Rev.

